# Delay of Aortic Arterial Input Function Time Improves Detection of Malignant Vertebral Body Lesions on Dynamic Contrast-Enhanced MRI Perfusion

**DOI:** 10.3390/cancers15082353

**Published:** 2023-04-18

**Authors:** Felipe Camelo, Kyung K. Peck, Atin Saha, Julio Arevalo-Perez, John K. Lyo, Jamie Tisnado, Eric Lis, Sasan Karimi, Andrei I. Holodny

**Affiliations:** 1 Department of Radiology, Memorial Sloan Kettering Cancer Center, New York, NY 10065, USA; 2 Weill Cornell Medicine, 1300 York Avenue, New York, NY 10065, USA; 3 Department of Medical Physics, Memorial Sloan Kettering Cancer Center, 1275 York Avenue, New York, NY 10065, USA; 4 Department of Radiology, Weill Medical College of Cornell University, 525 East 68th Street, New York, NY 10065, USA; 5 Department of Neuroscience, Weill Cornell Graduate School of Medical Sciences, 1300 York Avenue, New York, NY 10065, USA

**Keywords:** spine metastases, perfusion, dynamic contrast-enhanced MRI, arterial input function

## Abstract

**Simple Summary:**

Radiologists use dynamic contrast-enhanced MRI to study cancer in the vertebral bones. However, the current method assumes that the contrast material reaches the cancer cells at the same time as the surrounding blood vessels, usually using the aorta for measurement. This is not true for the vertebral bones because the contrast takes longer to reach the surrounding blood vessels in spinal cancers. To fix this problem, researchers shifted the curve of the contrast material and recalculated the values of contrast enhancement. They found that this new method showed cancer more accurately in the vertebral bones. This shows that radiologists need to carefully look at these MRI studies and adjust the contrast curve to obtain an accurate diagnosis.

**Abstract:**

Dynamic contrast-enhanced MRI (DCE) is an emerging modality in the study of vertebral body malignancies. DCE-MRI analysis relies on a pharmacokinetic model, which assumes that contrast uptake is simultaneous in the feeding of arteries and tissues of interest. While true in the highly vascularized brain, the perfusion of the spine is delayed. This delay of contrast reaching vertebral body lesions can affect DCE-MRI analyses, leading to misdiagnosis for the presence of active malignancy in the bone marrow. To overcome the limitation of delayed contrast arrival to vertebral body lesions, we shifted the arterial input function (AIF) curve over a series of phases and recalculated the plasma volume values (V_p_) for each phase shift. We hypothesized that shifting the AIF tracer curve would better reflect actual contrast perfusion, thereby improving the accuracy of V_p_ maps in metastases. We evaluated 18 biopsy-proven vertebral body metastases in which standard DCE-MRI analysis failed to demonstrate the expected increase in V_p_. We manually delayed the AIF curve for multiple phases, defined as the scan-specific phase temporal resolution, and analyzed DCE-MRI parameters with the new AIF curves. All patients were found to require at least one phase-shift delay in the calculated AIF to better visualize metastatic spinal lesions and improve quantitation of V_p_. Average normalized V_p_ values were 1.78 ± 1.88 for zero phase shifts (P0), 4.72 ± 4.31 for one phase shift (P1), and 5.59 ± 4.41 for two phase shifts (P2). Mann–Whitney U tests obtained *p*-values = 0.003 between P0 and P1, and 0.0004 between P0 and P2. This study demonstrates that image processing analysis for DCE-MRI in patients with spinal metastases requires a careful review of signal intensity curve, as well as a possible adjustment of the phase of aortic AIF to increase the accuracy of V_p_.

## 1. Introduction

Dynamic contrast-enhanced magnetic resonance imaging (DCE-MRI) is an emerging modality with the demonstrated ability to overcome some challenges in MRI of malignant spinal lesions. DCE-MRI noninvasively assesses tumor plasma volume (V_p_) and the permeability constant (K^trans^), which provide quantitative information about spinal lesions. DCE-MRI has been successfully applied to differentiate normal bone from tumor [1,2], malignant from benign vertebral compression fractures [3,4], and spinal metastasis from atypical vertebral hemangiomas [5], as well as to discriminate spinal metastases’ vascularity [6], identify the spinal metastases’ primary (renal cell versus prostate carcinoma) [7], accurately track post-radiation tumor response in chordomas [8], predict recurrence after radiation therapy [9], and quantitate changes in vertebral body metastases one hour post radiation therapy [10,11]. Recently, Guan et al. demonstrated that V_p_ derived from DCE perfusion could differentiate biopsy-proven malignant vertebral body lesions from non-neoplastic lesions with a sensitivity of 93%, specificity of 78%, and receiver operator characteristic curve of 88% [2]. 

Notwithstanding these successes, DCE of the spine remains an imperfect technique. For example, one hopes for higher specificity than 78% in differentiating malignant from non-malignant lesions [2]. While our institution has implemented more DCE-MRI for pathological spine cases, DCE-MRI analysis—initially developed for the brain—first appeared not to fully translate to the spine. The extended Tofts (ET) kinetic model is frequently used to derive pharmacokinetic parameters in DCE analysis [12,13,14]. This analysis depends upon the accurate selection of an arterial input function (AIF), which should reflect the flow of contrast through the end organ and the lesion itself. This estimation has proved to be reliable in DCE analysis of the brain due to high vascularity and short transit time between the arteries selected for AIF (typically the basilar or the MCA) and the brain [15]. The aorta is conventionally selected for AIF parameter estimation in the spine. However, there is a fundamental problem with this approach as ET assumes that the arterial contrast signal intensity curve is simultaneous to contrast uptake in vertebral body lesions. In fact, the bone marrow is much less vascular and is fed by smaller arteries than the brain is. Hence, the amplitude of perfusion in bone marrow is lower and the arrival of contrast is therefore delayed compared to the aorta. Recently, Liu et al., carried out a simulation to conceptually demonstrate the association between V_p_ and time delays in the AIF curve. They showed that V_p_ increased with increased time delays in the AIF in healthy individuals [16]. Therefore, V_p_ estimations made via AIF obtained with the assumption of simultaneous contrast uptake between the aorta and target lesion tissues may produce false negative results in spinal metastases.

Our study is motivated by Liu et al.’s findings of V_p_ association with time delay of the AIF tracer curve and our observation of clinical cases in which spine metastases did not demonstrate the expected increase in V_p_. We sought to study the relationship between V_p_ and various time delay shifts of AIF curves, as well as to evaluate the effects on V_p_ image quality and outcome comparison with histopathological findings. We hypothesized that shifting the aortic AIF tracer curve to more accurately reflect the delayed uptake time interval between the aorta and target lesion tissues would produce corrected V_p_ maps that showed higher correlation with the histopathological results.

## 2. Methods

### 2.1. Subjects

A total of 18 patients (12 male and 6 female) who underwent MRI perfusion studies of the spine were retrospectively analyzed. Consent was waived by IRB for the retrospective study. Neuroradiologists selected cases in which biopsy-verified, active metastatic disease on routine MR sequences did not correlate with the expected, concomitant increase in V_p_. These cases represent failures of DCE to correctly characterize active metastases. All cases were biopsy-verified for malignancy. 

### 2.2. Magnetic Resonance Imaging Acquisition

MR imaging of the spine was performed with a 3T GE scanner (Discovery 750W, GE Healthcare, Milwaukee, Brookfield, WI, USA) using an eight-channel cervical-thoracic-lumbar surface coil. Routine MR imaging sequences included: sagittal T1-weighted (field of view [FOV] = 32–36 cm; slice thickness = 3 mm; repetition time [TR] = 400–650 ms; flip angle [FA] = 90°), sagittal T2-weighted (FOV = 32–36 cm; slice thickness = 3 mm; TR = 3500–4000 ms; FA = 90°), and sagittal short tau inversion recovery (STIR) (FOV = 32–36 cm; slice thickness = 3 mm; TR = 3500–6000 ms; FA = 90°). Pre-contrast T1-weighted sagittal scan was acquired to match the perfusion images.

DCE perfusion was performed with a bolus of gadolinium-diethylenetriamine pentaacetic acid (Gd-DTPA) administered using a power injector at 0.1 mmol/kg and a rate of 2–3 mL/s. With the beginning of pre-injection time delay, kinetic enhancement of the tissue during and after injection of Gd-DTPA was obtained using a fixed spatial-temporal resolution Dixon imaging sequence (DISCO: Differential Subsampling with Cartesian Ordering) (TR = 4–5 ms; echo time [TE] = 1–2 ms; slice thickness = 5 mm; FA = 25°; acquisition matrix = 224 × 224 mm, field of view = 400 mm, pixel size = 1.78 mm × 1.78 mm, temporal resolution [Δt] = 3–4 s; phase volume = 60) and consisted of 12–14 images in the sagittal plane. Sagittal and axial T1-weighted post-Gd-DTPA images were acquired after perfusion imaging.

### 2.3. Preprocessing

Data were processed and analyzed using nordicICE version 4.2 (NordicNeuroLab, Bergen, Norway). Background noise removal was applied to remove the background noise and to analyze only the spine. Spatial smoothing with 2 mm Gaussian full width at half maximum (FWHM) and temporal smoothing were carried out to reduce high frequency noise and the spikes in dynamic signal response. The AIF was selected from the aorta at the level closest to the metastatic lesion, where the dynamic curve exhibited a rapid increase in signal enhancement, showing a sharp peak followed by minimal temporal noise, and adequate washing out after the peak. Deconvolution with the AIF was performed to estimate perfusion parameters. ET two-compartment pharmacokinetic model was applied to calculate perfusion parameter V_p_. Regions of interest (ROI) were manually selected and included both metastatic foci and normal bone marrow. 

To account for background variations among DCE-MRI perfusion scans, plasma volume (V_p_) was normalized by obtaining the ratio between lesion ROI and local neighboring healthy vertebra (V_p_ normalization = V_p_ of lesion/V_p_ of normal bone). The normalized V_p_ values were used for statistical analyses.

### 2.4. AIF Tissue Delay and Shift of Aorta Response

The ET model assumes rapid flow relative to the sampling rate (phase temporal resolution) of the sequence so that the transit time of the contrast through tissue is essentially immediate. Under these conditions, the total concentration of contrast (*C_t_*) in the tissues can be given as follows:

(1) $$C_{t}\left(t\right)=v_{p}C_{p}\left(t\right)+C_{p}\left(t\right)\;⨂K^{trans}\exp\left(-K^{trans}t/v_{e}\right)$$

where *C_t_* is the total tissue contrast agent (CA) concentration, *V_e_* is the extravascular extracellular volume, *t* is time, and *C_p_* is the CA concentration in plasma, determined from a well-defined artery feeding the tissue of interest, which is the AIF. 

The ET kinetic model assumes that the AIF and tissue contrast onset occur simultaneously. However, there may be a significant delay between the arrival of contrast in the aorta and in the lesion. Additionally, this difference varies between tissue types, normal and pathologic tissues, and individual patients. Our study observed a significant contrast onset delay between the aorta and bone marrow lesions in some patients, which resulted in the underestimation of V_p_, thereby creating false negative outcomes. To address this delay, the signal intensity curve in the aorta was manually shifted, depending on the degree of the delay. In the current study, four different phase-shift delays (from zero to three phases) were applied, along with qualitative and quantitative analysis of associated V_p_ maps. 

### 2.5. Statistical Analyses

A Mann–Whitney U test (at a significance level of *p* ≤ 0.01) was conducted to assess the difference between V_p_ obtained with various phase-shift delays.

## 3. Results

All 18 evaluated patients (Table 1) were found to require at least one phase-shift delay in the calculated AIF to improve accuracy in quantification of V_p_, which, in turn, contributes to a more accurate visualization of the metastasis on overlay maps with anatomical images. 

**Figure 1:**
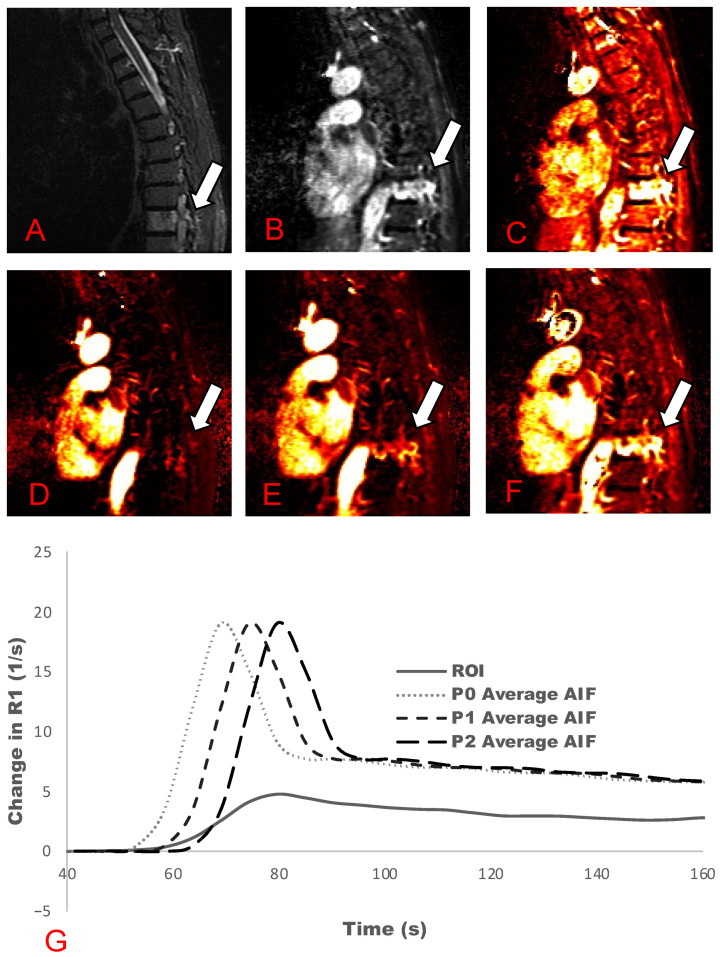
Active metastasis in T9 (Table 1: Patient 10). Multiple phase-shift delays and associated plasma volume (V_p_) maps are presented. (**A**) STIR map; (**B**) Contrast uptake map; (**C**) K^trans^ map; (**D**) V_p_ maps without a phase-shift delay; (**E**) one phase-shift delay; and (**F**) two phase-shift delays. (**G**) AIF graph demonstrating the phase-shift delay at zero (P0), one (P1), and two (P2) phase shifts of the average AIF curve. Solid line demonstrates lesion ROIs.

**Figure 2:**
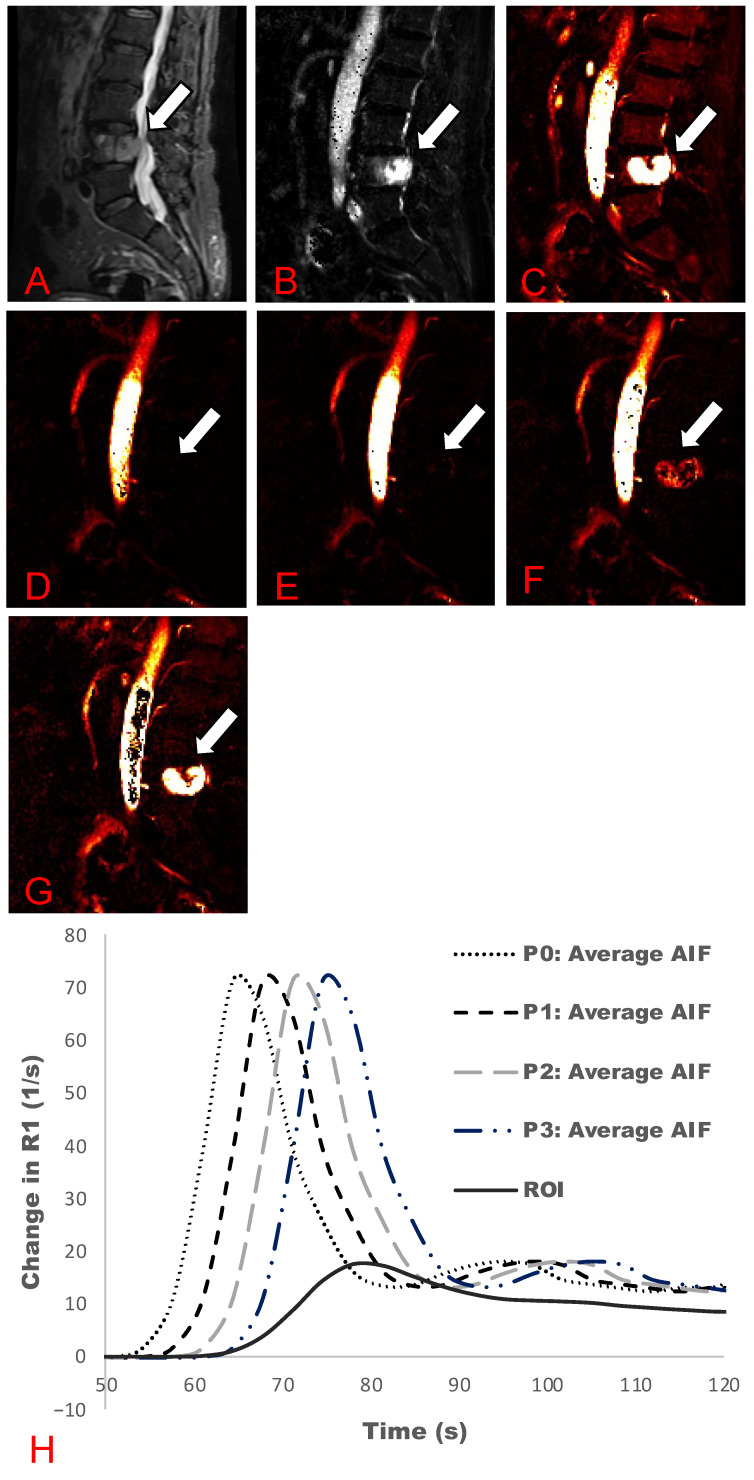
Active metastasis in L4 (Table 1: Patient 13). Multiple phase-shift delays and associated plasma volume (V_p_) maps are presented. (**A**) STIR map; (**B**) Contrast uptake map; (**C**) K^trans^ map; (**D**) V_p_ maps without a phase-shift delay; (**E**) one phase-shift delay; (**F**) two phase-shift delays; and (**G**) three phase-shift delays. (**H**) AIF graph demonstrating the phase-shift delays at zero (P0), one (P1), two (P2), and three (P3) phase shifts of the average AIF curve. Solid line demonstrates the lesion ROIs.

Figure 1 and Figure 2 demonstrate a comparison between T2-weighted MRI images with and without contrast uptake, and DCE analyses for K^trans^ and V_p_ maps for phase shifts from zero to three. Across all cases, increasing the time delay (phase shifts) led to an increased V_p_ value and visual hyperintensity on V_p_ maps (Figure 1D–F and Figure 2D–G). Signal hyperintensity of the ROI, AIF, and manually delayed AIF curves were graphed to compare peak hyperintensities over time (Figure 1G and Figure 2H). These graphs demonstrate that ROI peak signal hyperintensity corresponds better with the peaks of manually delayed AIF curves. 

**Figure 3:**
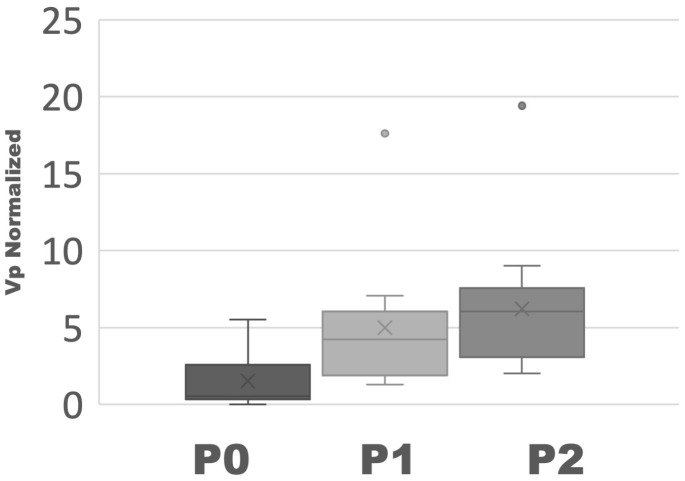
Box and whisker plot of V_p_ (*n* = 18). One circle in P1 and one circle in P2 are represented as outliers.

The average V_p_ values (*n* = 18) were 1.78 ± 1.88 for zero phase shifts, 4.72 ± 4.31 for one phase shift, and 5.59 ± 4.41 for two phase shifts (Figure 3). Mann–Whitney U tests were performed and obtained *p*-values of 0.003 between P0 and P1, and of 0.0004 between P0 and P2.

## 4. Discussion

In this study, we demonstrated that manual phase-shift delays of the AIF signal intensity curve in cases of spinal metastases whose standard analysis yielded suboptimal perfusion results can increase the diagnostic accuracy of perfusion parameters. These findings are concordant with the results of Liu et al., whose controlled simulations showed that V_p_ increases as AIF delay time increases in healthy individuals [16]. 

In the brain (the organ in which perfusion is most performed), perfusion can be assumed to be simultaneous in the artery, vein, and brain parenchyma. Filice and Crisi demonstrated that direct comparison of DCE-MRI measurements with AIF generated by means of arterial or venous waveform in high-grade glioma patients led to no statistical difference in quantitative metrics [15]. However, perfusion of the spine fundamentally differs. While the aorta is usually selected for definition of the AIF, actual perfusion of the bone marrow is accomplished through tiny arteries. Additionally, the volume of perfusion of the brain is very high, whereas the perfusion of bone marrow (especially in older individuals) is much lower [8]. Ideally, AIF should be sampled at the vascular inlet of the target tissue. Liu et al. suggested using spine segmental arteries for V_p_ analyses instead of the aorta [16]. However, sampling the tiny arteries that feed bone marrow is impractical due to their being difficult to identify, highly variable, and of noisy signal intensity. Our study used sagittal slices for dynamic perfusion acquisition, rendering AIF measurement with vertebral segmental arteries unfeasible due to limited spatial resolution. Lui et al. selected axial slices, which allowed them to identify and quantify lumbar perfusion, but prevented visualization of multiple vertebral bodies during a single scan, and presented limitations due to volume and flow effects. Using spinal segmental arteries on axial images to measure the AIF, however, is not practical for daily clinical practice due to various factors including the potentially time intensive nature of the process, necessity for patient compliance, and inability to evaluate more than one level at a time. 

Optimization of the technical aspects of spinal DCE-MRI remains a challenge due to physical and physiological limitations. Whole spine imaging quality, particularly of the thoracic area, is affected by physiological phenomena, such as breathing and heartbeat, resulting in motion artifact. AIF measurements are also affected by flow, whose effects can be minimized with the use of shorter TE and TR. However, these adjustments come at the expense of a lower signal-to-noise ratio, making conventional MRI vertebral body analysis more challenging [17]. We therefore believe that manually delaying the AIF curve could be a very useful and relatively simple technique to improve the diagnostic accuracy of DCE-MRI perfusion in the spine.

Our study’s limitations include its small patient population, as well as the nonrandom selection of participants that were observed to have conflicting DCE-MRI results from conventional MRI. Further investigation into specific patient parameters that indicate a potential benefit from AIF phase-shift delay is required. Further, this study considered only spinal metastases and did not consider other pathologies often difficult to differentiate from vertebral metastases, such as atypical hemangiomas or acute vertebral body fractures. We also did not investigate each individual case to evaluate if cardiac output, blood pressure, age, gender, type of primary malignancy, or other specific physiological factors might contribute to delayed AIF. Although previous studies suggest that V_p_ is sufficient or often superior for the diagnosis of spinal lesions, we did not evaluate the effects of AIF time delay on other DCE-MRI parameters [3,4,5,9,10].

## 5. Conclusions

Based on this study’s findings, radiologists and technologists should take this phenomenon of tissue contrast uptake delay into consideration, especially when images from V_p_ maps are not concordant with suspicious conventional MRI findings. It is important that both analysts and software developers be aware of pitfalls that erroneously increase the number of false negative perfusion results. Manually delaying the AIF curve to simulate the delay between the aorta and the capillaries within the bone marrow could be a potential solution to improve V_p_ and reduce false negative results.

## Figures and Tables

**Table 1:** Patient demographics.

Patient	Gender	Age	Primary Cancer	Location	Pretreatment from Perfusion Date
1	F	69	Cholangiocarcinoma	L3	Chemotherapy
2	M	55	Breast Cancer	T10 T11, (previous L2 metastases)	Chemotherapy
3	M	62	Renal Cell Carcinoma	T12, L3	Chemotherapy
4	F	71	Lung Adenocarcinoma	C7	None
5	M	75	Multiple Myeloma	T6, T7, T12, L1	Chemotherapy
6	M	63	Cholangiocarcinoma	L3, L4	Chemotherapy
7	M	53	Colon Cancer	L2 L3	Chemotherapy
8	F	50	Colon Cancer	L3	Chemotherapy
9	M	80	Myoepithelioma	S2	RT two years prior
10	F	63	Thymoma	T9	Resections, RT, Chemo
11	F	50	Renal Cancer	T12	None
12	F	53	NSCLC	L2	none
13	M	74	Thymoma	L4	None
14	M	55	Melanoma	L2, L3	Immunotherapy
15	M	63	Prostate	T5 and Sacrum	None
16	M	62	Colon Cancer	L5-S3	None
17	F	61	NSCLC	T7 T8	Chemotherapy
18	M	37	Renal Cancer	L4	None

## Data Availability

The data are not publicly available due to the sensitive nature of the data that includes private health data and imaging.
